# Experimental Study on the Mechanical Properties and Microstructural Mechanisms of Coal Gangue-Based Cementitious Materials Synergistically Activated by Desulfurization Gypsum and Lime

**DOI:** 10.3390/polym17070932

**Published:** 2025-03-29

**Authors:** Luyao Wang, Qingping Wang, Jingyi Cai, Feifei Zhou, Zhiwei Cheng, Jiayao Zhang

**Affiliations:** 1 School of Materials Science and Engineering, Anhui University of Science and Technology, Huainan 232001, China; 18655792876@163.com (L.W.); cjy960628@163.com (J.C.); ffzhou@aust.edu.cn (F.Z.); czwxym@163.com (Z.C.); z17352180500@163.com (J.Z.); 2 State Key Laboratory of Mining Response and Disaster Prevention and Control in Deep Coal Mines, Anhui University of Science and Technology, Huainan 232001, China; 3 Anhui Industrial Generic Technology Research Center for New Materials from Coal-based Solid Wastes, Huainan 232001, China

**Keywords:** coal gangue, flue gas desulfurization gypsum, lime, activation, cementitious materials, environmentally friendly

## Abstract

Coal gangue and flue gas desulfurization gypsum (DG) are industrial by-products whose utilization is critical for sustainable development. This study explores the synergistic activation of coal gangue using DG and lime to develop eco-friendly cementitious materials. Three systems—CF (coal gangue–DG), CL (coal gangue–lime), and CFL (coal gangue–DG–lime)—were designed to investigate the effects of DG and lime on coal gangue reactivity. Unconfined compressive strength tests identified the optimal dosages of DG and lime, while XRD, SEM, TGA-DTG, and NMR analyses were employed to characterize the hydration products and microstructure. The results demonstrated that calcium ions from both lime and DG facilitated the formation of C-(A)-S-H gel, enhancing strength development. However, excessive DG led to undesirable volume expansion due to ettringite overproduction, compromising material stability. Excess lime forms weak Ca(OH)_2_ binding phases and causes expansion and cracking from excessive hydration heat, reducing strength and durability. Thus, the optimal DG dosage in the CF system is 70%, and the optimal lime dosage in the CL system is 6%. In the CFL system, 70% DG and 4% lime achieve the best performance. The combined use of DG and lime not only improved coal gangue reactivity but also achieved an optimal balance between strength enhancement and long-term stability, offering a promising approach for sustainable construction materials.

## 1. Introduction

China’s abundant mineral resources have significantly contributed to its rapid economic development. However, the extraction of these resources generates substantial amounts of industrial by-products, such as coal gangue and flue gas desulfurization gypsum (DG). Coal gangue, which is a by-product obtained from coal mining and washing operations, has a low carbon content, is hard, and is difficult to burn, leading to its low utilization rate [1,2].

Currently, China produces approximately 750 million tons of coal gangue annually, yet its utilization rate remains below 50%, resulting in large-scale accumulation and environmental hazards. DG is generated during flue gas desulfurization in coal-fired power plants to reduce SO_2_ emissions [3]. Despite its chemical compositions is similar to natural gypsum, DG has a complex composition, a high decomposition temperature, and a low recycling rate, with most of it being stored as solid waste [3,4,5].

In 2023, China’s industrial solid waste was 3.97 billion tons, with coal gangue accounting for 18.7% [6]. Long-term accumulation of coal gangue at mining sites leads to the formation of gangue heaps, which pose serious environmental problems, including spontaneous combustion, acid mine drainage, and heavy metal leaching. These environmental risks not only degrade surrounding ecosystems but also contribute to air and water pollution, threatening public health and sustainable development.

Coal gangue’s rich chemical and mineral composition gives it significant resource utilization potential [7]. Its primary uses include building materials, power generation, soil improvement, and mineral extraction [8]. However, its low reactivity and complex composition have limitation of its performance in these areas, and its high processing costs results in its low overall utilization rate, which remains below 50% [9]. Large accumulations of coal gangue at mining sites form gangue heaps, which pose serious environmental problems, such as spontaneous combustion, acid mine drainage, and air and water pollution [10].

Similarly, the production of DG continues to increase, and by 2023, it had become one of the major industrial by-products of coal-fired power plants [11]. DG, composed primarily of calcium sulfate dihydrate (CaSO_4_·2H_2_O), shares properties with natural gypsum and holds potential for resource utilization [12]. Current applications of DG are mostly in building materials, such as cement hydration retarders and concrete additives, as well as in gypsum board [13]. However, due to its high moisture content and the potential risk of heavy metal leaching, the large-scale utilization of DG remains a challenge [14]. Improper disposal of DG leads to land occupation, dust emissions, and contamination of soil and groundwater, exacerbating environmental concerns [15]. Comprehensive management and utilization of DG thus face significant challenges.

The resource utilization of coal gangue currently relies on three activation methods: physical, thermal, and chemical, all of which face limitations, such as high energy consumption, high costs, and low efficiency [16]. Coal gangue contains reactive silicon dioxide (SiO_2_) and aluminum oxide (Al_2_O_3_); however, its dense structure, high glass phase content, and low hydraulic activity hinder its cementitious potential [17]. Traditional methods, such as utilizing coal gangue in the capacity of a supplementary cementitious material or lightweight aggregate, result in low-value applications and fail to maximize its potential [18]. Therefore, innovative and high-value utilization strategies are needed to enhance the reactivity of coal gangue through activation, enabling it to serve as a primary cementitious material [19].

Physical activation methods, like fine grinding and ultrasonic treatment, increase coal gangue’s surface area and particle fineness to improve reactivity, though these methods are costly and provide limited improvement [20]. Thermal treatment, involving high-temperature calcination (600–900 °C), enhances the reactivity of coal gangue by converting the kaolinite within it into metakaolin through the dehydroxylation process [21]. While effective, thermal treatment is energy-intensive and less suitable for low-carbon applications. In contrast, chemical activation using external activators like lime or sulfates enhances coal gangue’s reactivity under low temperatures [22]. Lime (CaO), a common activator, reacts with the alumina and silica in coal gangue to form cementitious products like C-S-H and C-A-H, improving both the material’s strength and durability [23,24]. However, lime activation alone is associated with delayed hydration, leading to lower early strength development and limiting its practical application in time-sensitive construction materials [25].

Recent pieces of research have explored the potential of DG as an activator in coal gangue-based cementitious systems [25,26]. In an alkaline environment, DG releases SO_4_^2−^ ions, which react with Ca^2+^ and Al^3+^ ions in CG to form AFt and CSA, promoting early strength development [27]. The incorporation of DG improves the flowability and compactness of cementitious materials, enhancing overall performance [28]. However, DG’s low binding ability limits its effect when used alone. To address this limitation, researchers have investigated the combination of DG with other activators (such as lime) to develop a multi-component system that leverages the synergistic effects of both components [29]. Despite these efforts, the underlying hydration mechanisms and the influence of different FDG-to-lime ratios on coal gangue activation remain inadequately understood [30].

Despite the promising potential of DG–lime activation systems, current research has not fully elucidated the combined effects of DG and lime on coal gangue activation, particularly in terms of hydration mechanisms, microstructural evolution, and mechanical performance [31]. In Moreover, existing studies have not systematically evaluated the impact of varying replacement ratios on setting time, flowability, and strength development [32]. To bridge these knowledge gaps, this study investigated the combined use of FDG and lime in coal gangue-based cementitious systems, focusing on their effects on hydration mechanisms and cementitious properties [33].

Based on the above background, in this study three coal gangue activation systems were designed: CF (coal gangue–desulfurized gypsum), CL (coal gangue–lime), and CFL (coal gangue–desulfurized gypsum–lime). To explore the synergistic effects of FDG and lime in coal gangue activation, X-ray diffraction (XRD), scanning electron microscopy (SEM), thermogravimetric analysis (TGA-DTG), and nuclear magnetic resonance (NMR) were employed to systematically analyze the microstructure and the evolution of hydration products in the three systems. In addition, this study investigates the effects of different replacement ratios on setting time, flowability, and uniaxial compressive strength. The results provide new insights and technical approaches for the high-value utilization of coal gangue, contributing to both resource efficiency and environmental sustainability.

## 2. Materials and Methods

### 2.1. Raw Materials

#### 2.1.1. CG, DG, and Lime

**Figure 1:**
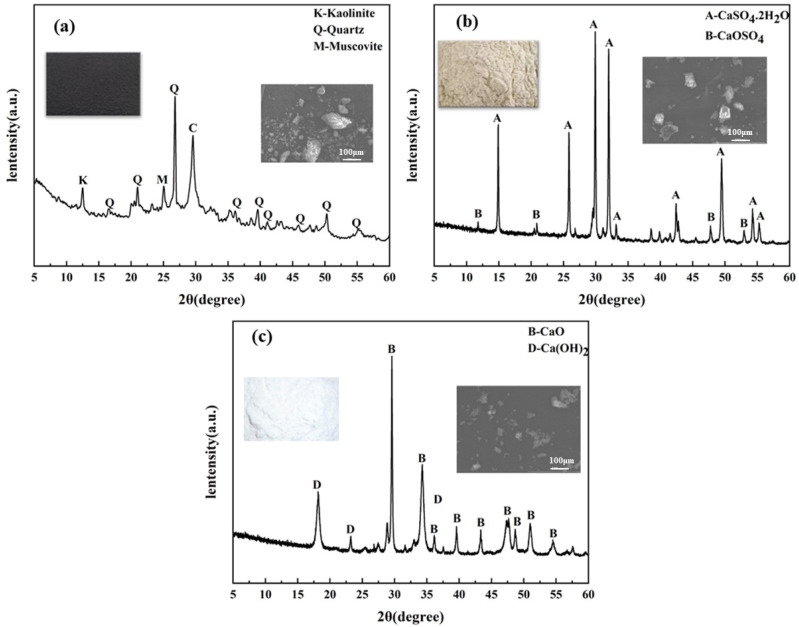
XRD of three raw materials: (**a**) CG, (**b**) DG, and (**c**) lime.

The main raw materials in this study were CG, DG, and lime. Their micro-morphologies and compositions are shown in Figure 1 and Table 1. As seen in Figure 1, CG mainly contains quartz, kaolinite, and calcite. DG’s main component is CaSO_4_·2H_2_O, and lime mainly consists of CaO with a bit of Ca(OH)_2_.

**Figure 2:**
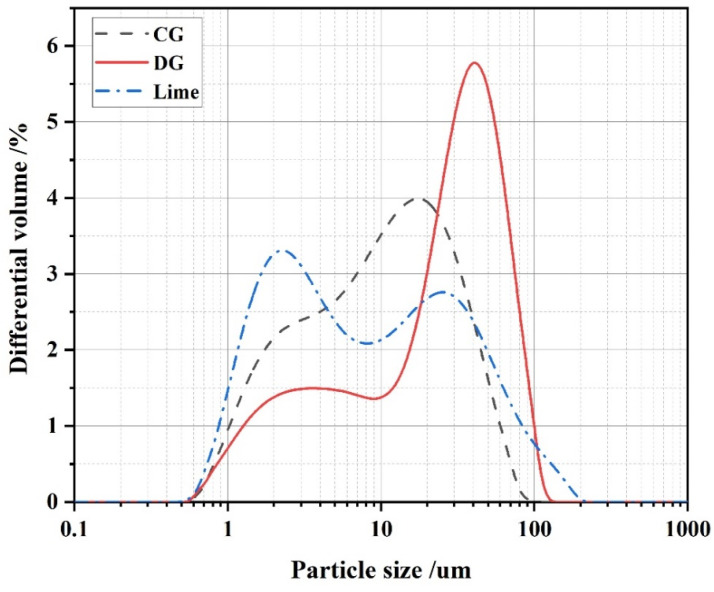
Distribution of particle sizes of CG, DG, and lime.

Table 2 and Figure 2 reflect the laser particle size test results of the three raw materials, with the median particle sizes (D_50_) of CG, DG, and lime being 9.674 μm, 25.605 μm, and 8.022 μm, respectively. In addition, Table 2 shows that the powder’s numerical dispersion is less than 10 μm, suggesting good gradation of raw materials beneficial to the stability of cementitious materials’ performance [34].

#### 2.1.2. Pretreatment of DG

**Figure 3:**
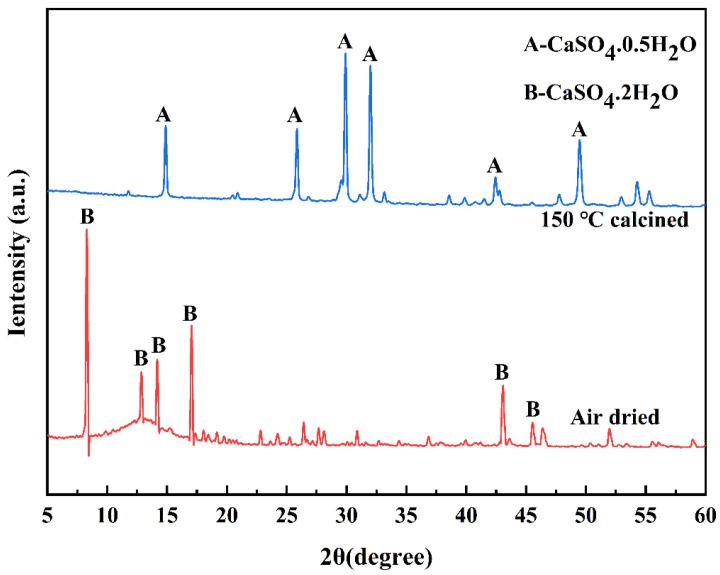
XRD patterns of the air-dried DG and the DG calcined at 150 °C for 2 h.

To activate the potential reactivity of coal gangue, gypsum is typically pretreated by converting CaSO_4_·2H_2_O into CaSO_4_·0.5H_2_O [35,36]. CaSO_4_·2H_2_O is chemically stable and exhibits a slow hydration reaction rate, making it challenging to effectively activate the reactive mineral components in coal gangue when used in its natural form [37]. Therefore, previous studies have investigated the effects of calcining dihydrate gypsum at various temperatures (150 °C, 250 °C, 350 °C and 450 °C) on the strength of composite systems. The results indicate that gypsum calcined at 150 °C leads to higher early-age strength, whereas calcination at 450 °C or higher results in a reduction in strength due to the formation of anhydrous gypsum [38]. Consequently, calcination at 150 °C for 2 h has been identified as the optimal pretreatment method. The CaSO_4_·0.5H_2_O produced through this process exhibits enhanced surface reactivity, which significantly improves the mechanical properties of coal gangue-based composites, particularly in terms of early-age strength development. The XRD patterns of air-dried and 150-calcined gypsum are shown in Figure 3, and the results indicate that the primary function of pretreatment is to remove a portion of the crystal water from CaSO_4_·2H_2_O, thereby transforming it into the more reactive CaSO_4_·0.5H_2_O phase.

### 2.2. Experimental Design

First, the individual activation effects of desulfurized gypsum and lime were evaluated in the CF and CL systems, optimizing their dosages and revealing their interaction mechanisms with coal gangue. Based on these results, the ternary CFL system was designed to explore the synergistic effects of desulfurized gypsum and lime, focusing on their combined influence on the mechanical properties of cementitious materials.

The experiment was divided into three parts, each corresponding to one of the systems, as shown in Table 3.

(1) CF System (Coal Gangue–DG): in this system, DG was used to partially replace CG at mass ratios ranging from 10% to 90%, with 10% increments for each gradient.
(2) GL System (Coal Gangue–Lime): here, lime was used to replace coal gangue in proportions from 2% to 10%, with 2% increments.
(3) CFL System (Coal Gangue–DG–Lime): Based on the results of the CF and CL systems, the replacement level of desulfurized gypsum was fixed at 70%. Lime was added in varying amounts (1% to 6%, with 1% increments) to study its effect on the material properties.

### 2.3. Preparation of Samples

**Figure 4:**
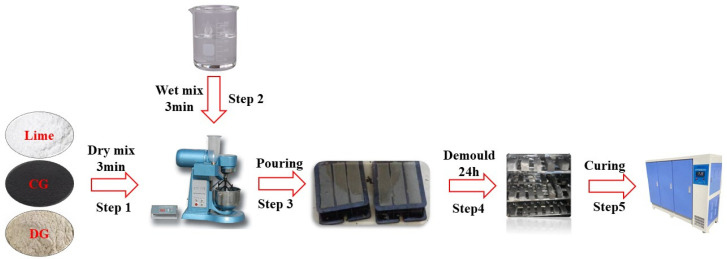
Sample preparation procedures.

Samples were prepared with a laboratory slurry mixer. Firstly, CG, DG, and lime were dry-mixed for 3 min to guarantee uniform component distribution. Subsequently, water was added and the mixture was stirred for another 3 min to obtain a homogeneous slurry. The slurry was then poured into 40 mm × 40 mm × 160 mm cubic molds for strength testing. After casting, the samples, along with the molds, were placed in a curing room maintained at 20 ± 2 °C and 95 ± 5% relative humidity. The samples were demolded after 24 h and subsequently cured under the same conditions for 3, 7, and 28 days. The detailed sample preparation process is illustrated in Figure 4.

### 2.4. Test Methods

#### 2.4.1. Particle Size Analysis

The granulometric distributions of these materials were measured by an EyeTech laser diffraction particle size analyzer (Mastersizer 3000+ Ultra, Malvern Panalytical, Worceste, UK) within 0.1–100 μm.

#### 2.4.2. Unconfined Compression Test

The unconfined compression test was carried out by the Chinese standard [39]. To ensure the precision pertaining to the measurements, three samples of each mixture were tested, and the results were computed as an average.

#### 2.4.3. XRD Test

To analyze the mineral components within the paste, X-ray diffraction (XRD-600, Shimadzu Instrument Co., Ltd., Kyoto, Japan) was employed. The XRD apparatus was operated using a Cu-Ka radiation source. The XRD data were gathered through scans in the angular range from 5° to 80°.

#### 2.4.4. SEM Test

The microstructure of the hydration products was observed using The Scanning Electron Microscope (Flex SEM 1000, Shanghai Chenhua Instrument Co., Ltd., Shanghai, China), which has an accelerating voltage range of 15.0 kV, was utilized to capture the morphology of the sample.

#### 2.4.5. TGA-DTG

TGA measurements were carried out on a Mettler Toledo TGA2 instrument (Mettler Toledol, Columbus, OH, USA). Around 10–20 mg of finely ground sample was put into a platinum crucible. The temperature was increased from room temperature to 1000 °C at a 10 °C/min heating rate under a nitrogen atmosphere.

#### 2.4.6. NMR Test

NMR experiments were conducted on a Bruker Ascend Evo 1.0 GHz NMR spectrometer (Bruker Corporation, Billerica, MA, USA). In solid-state NMR, samples were loaded into a 4 mm zirconia rotor and spun at 25 kHz for magic-angle spinning (MAS). The ^29^Si NMR spectra were obtained via standard pulse sequences, with chemical shifts referenced to TMS as an internal standard.

## 3. Testing Results

### 3.1. The Effect of the CF System on the Compressive Strength

**Figure 5:**
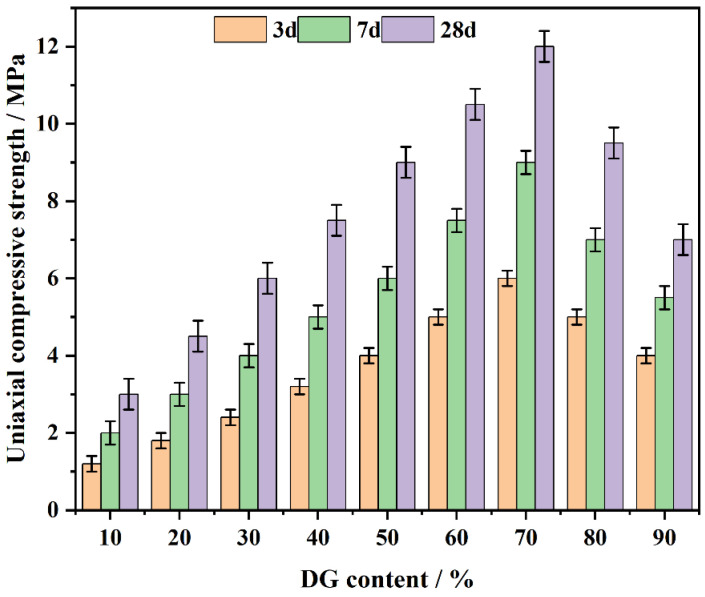
The effect of DG on the CF system’s compressive strength over days.

The compressive strength of the CF system was subjected to testing at curing ages of 3, 7, and 28 days (Figure 5). The experimental outcomes show an overall upward trend in compressive strength as DG content increases, reaching a maximum at 70% DG, and then a subsequent decrease beyond this point. This phenomenon can be ascribed to the initial generation of CaSO_4_·2H_2_O, which functions to fulfill pore spaces and the subsequent formation of C-S-H gel that serves as the predominant contributor to the strength in the later phase.

At DG contents between 10% and 30%, the concentration of available Ca^2+^ ions is insufficient to fully support the hydration reactions, leading to a limited formation of gypsum crystals and reduced compressive strength due to poor pore-filling effects. This insufficient Ca^2+^ concentration explains the lower strength observed in this range, as the material struggles to form sufficient hydration products.

As the DG content increases to 40–70%, the hydration products significantly increase, reaching their peak at 70%, where gypsum and C-S-H combine to form the densest microstructure [40]. The resulting internal structure improves both the early and long-term compressive strength of the system.

Beyond 70% DG content, the observed strength decrease can be attributed to an excess of gypsum, which forms weaker binding phases with free sulfate ions and unreacted DG. This leads to a more brittle structure and reduced strength at later curing ages. At higher DG levels, the overabundance of unreacted gypsum disrupts the optimal hydration process, weakening the material’s overall integrity. Additionally, as the curing age progresses to 28 days, the active coal gangue components react with Ca^2+^ to form additional C-S-H and AFt, which further enhances strength [41].

Thus, at 70% DG, the CF system exhibits the optimal balance of hydration products, achieving the highest compressive strength. This balance is due to the enhanced formation of gypsum dihydrate in the early stages and the ongoing production of C-S-H gel and AFt, which contribute to long-term strength [42]. In contrast, excessive DG content results in poorer structural integrity due to an overabundance of unreacted gypsum, which disrupts the formation of stable hydration products.

### 3.2. The Effect of CL System on the Compressive Strength

**Figure 6:**
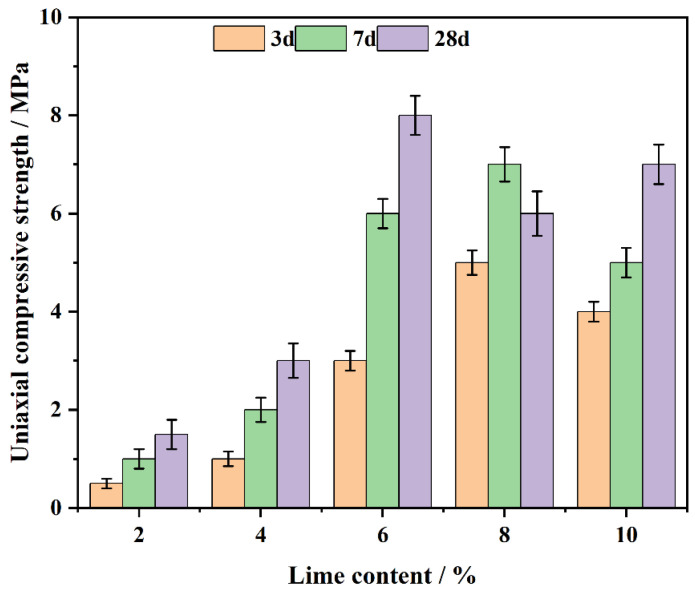
The effect of lime on the CL system’s compressive strength over days.

The CL system’s compressive strength was evaluated at curing ages of 3, 7 and 28 days (Figure 6). The results show that the compressive strength increases with lime content up to 6%, reaching a peak, and then decreases with further increases in lime content. The strength development results from the prior formation of Ca(OH)_2_ and the later emergence of C-S-H gel [43].

At lime contents between 2% and 4%, the available Ca^2+^ [44] ions are insufficient to fully support hydration reactions, leading to limited Ca(OH)_2_ formation and poor pore filling. As the lime content increases to 6%, the hydration substances generated, including Ca(OH)_2_ and C-S-H, are maximized, resulting in a dense microstructure and the highest compressive strength.

However, when the lime content exceeds 6%, the excess Ca^2+^ ions cannot effectively participate in further pozzolanic reactions with coal gangue. This leads to the formation of weak binding phases, as the unreacted Ca(OH)_2_ creates [45] a more porous and brittle matrix. Excessive lime also introduces unreacted particles, which disrupt the microstructure, increasing the pore volume and reducing the overall strength of the system.

Additionally, at 28 days, the remaining reactive components in coal gangue contribute to the formation of additional C-S-H [46], which further enhances the system’s strength. However, in systems with excessive lime (8% or more), the presence of unreacted Ca(OH)_2_ leads to micro-cracks and structural brittleness, reducing long-term strength. This occurs because excess Ca(OH)_2_ fails to participate fully in the pozzolanic reaction, resulting in weak bonding phases that compromise overall structural integrity. As the curing process continues, the effects of unreacted Ca(OH)_2_ are exacerbated, leading to localized stress concentrations and further weakening the material. Additionally, the heat released during hydration and changes in moisture levels can induce volumetric expansion and cracking, ultimately hindering the durability of the system.

Thus, the system with 6% lime content achieves the optimal balance, providing the optimal early-stage and long-term compressive strength. The formation of sufficient Ca(OH)_2_ early on fills pores and contributes to initial strength, while the ongoing production of C-S-H increases strength over time. In contrast, lime contents beyond 6% result in a detrimental effect on the material’s performance due to excessive unreacted lime and weak matrix formation, leading to a decline in compressive strength.

In summary, an optimal lime content of 6% provides the CL system with superior compressive strength by achieving the right balance between Ca(OH)_2_ and C-S-H formation. Excessive lime, however, leads to the formation of weak binding phases, increasing porosity and reducing strength [47].

### 3.3. The Effect of CFL System on Compressive Strength

**Figure 7:**
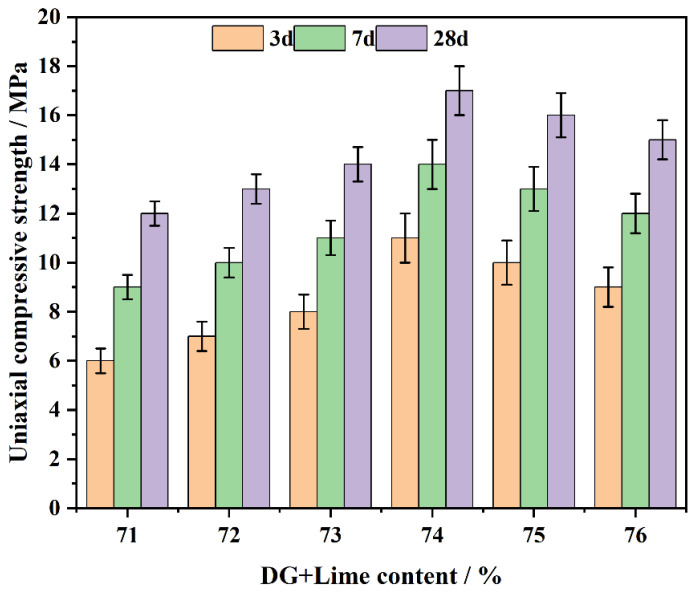
The impact of DG + lime on the CFL system’s compressive strength over days.

The CFL system’s compressive strength was assessed with 70% DG content and 1–6% lime content. Experiments were conducted at curing ages of 3, 7 and 28 days (Figure 7). The results indicate that the compressive strength first ascends with the combined DG and lime content, peaks at 74%, and subsequently declines upon further increase of the content.

The initial rise of compressive strength is ascribed to the synergistic generation of C-S-H gel, CaSO_4_·2H_2_O and Ca(OH)_2_. In the early stages, CaSO_4_·2H_2_O and Ca(OH)_2_ contribute [47] to filling pores and forming a solid network structure, providing early strength. As the system continues to cure, C-S-H gel becomes the dominant phase, enhancing long-term strength.

When the combined content of DG and lime is between 71% and 73%, the compressive strength remains lower compared to the 74% content, as observed in the data. The concentration of Ca^2+^ ions in this range may not be sufficient to maximize the formation of Ca(OH)_2_ and CaSO_4_·2H_2_O, which correlates with the observed lower compressive strength values. In contrast, at a combined content of 74%, the data shows a significant increase in hydration products, leading to the formation of a denser microstructure, which is associated with improved compressive strength. At this level, the balance between Ca(OH)_2_ and CaSO_4_·2H_2_O crystals is optimal, resulting in the maximum compressive strength during both the early and later periods.

Beyond 74% combined DG and lime content, the excess lime and DG cannot effectively engage in pozzolanic reactions. This results in the development of weak binding phases, as the free Ca^2+^ [48] ions interact with unreacted components, leading to a more porous and brittle matrix. This weakens the system’s structural integrity and reduces compressive strength, particularly at later curing ages.

Additionally, as curing progresses, particularly at 28 days, the active components in coal gangue participate in secondary reactions, further reacting with Ca^2+^ ions to form additional C-S-H and (ettringite) [49]. These reactions improve the overall strength. However, in cases where the DG and lime content is too high, the resulting matrix is brittle, and the excess unreacted material prevents further strength development. Therefore, the system with 74% combined DG and lime content shows the maximum and most consistent compressive strength, with superior strength at both early and later curing stages.

Compared with other mix proportions, the system with 74% combined DG and lime content demonstrates significant early-stage strength advantages (at 3 and 7 days). This is because moderate levels of DG and lime accelerate the formation of Ca(OH)_2_ and CaSO_4_·2H_2_O, which quickly fills pores and provides initial structural support. As curing continues, the stable concentration of Ca^2+^ ions promotes the formation because of the presence of supplementary C-S-H gel and AFt, leading to a further increase in strength.

For CFL system, the variation in the combined DG and lime content and curing age significantly influences hydration products and pore structure evolution, both of which directly affect compressive strength. An optimal combined content of 74% DG and lime provides the best balance between early and long-term strength development. Conversely, elevated DG and lime content induces the formation of weakly binding phases and strength reduction.

### 3.4. The Effect of DG and Lime Dosages on the Sample Density

**Figure 8:**
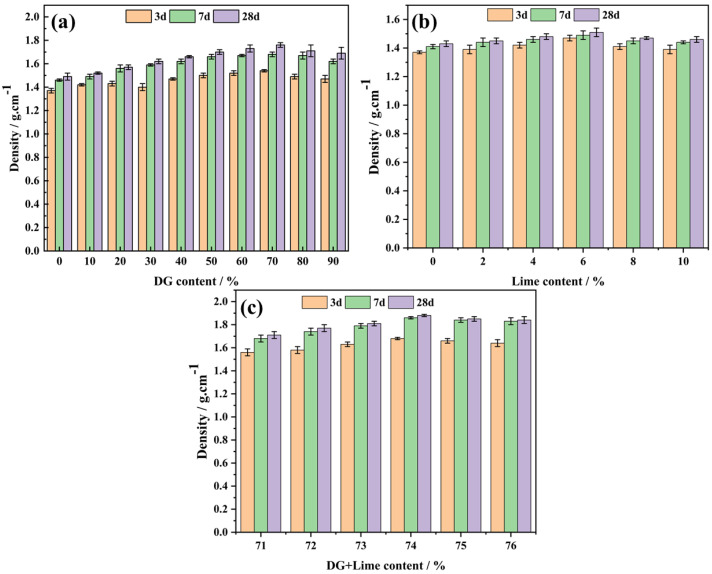
The effect of DG and lime dosages on the sample density over days: (**a**) CF system, (**b**) CL system, and (**c**) CFL system.

Figure 8 presents the density variations of the CF, CL, and CFL systems with different DG and lime dosages at various curing ages. These findings complement the compressive strength results discussed earlier, providing a comprehensive understanding of the material’s properties.

In the CF system (Figure 8a), the density initially increases with DG content, reaching a maximum of 70%, then decreases. This trend aligns with the strength development, where optimal strength coincides with peak density. The density increase is attributed to DG particles filling pores within the coal gangue, creating a more compact structure. However, beyond 70% DG, the density declines, likely due to excess DG introducing new pores or inadequate bonding with coal gangue.

For the CL system (Figure 8b), the density rises to a peak at 6% lime content and then diminishes. This corresponds to the strength variation, where maximum strength occurs at 6% lime. At lower lime contents, insufficient Ca^2+^ limits pore filling by Ca(OH)_2_. Excess lime beyond 6% forms weak binding phases and increases porosity, reducing density.

In the CFL system (Figure 8c), the density peaks at 74% combined DG and lime content, consistent with the strength results. This combination optimizes the formation of hydration products, resulting in a dense microstructure. Excess DG and lime lead to weak phases and higher porosity, decreasing density.

Overall, the density trends in all systems correlate with their compressive strength developments. Optimal DG and lime dosages enhance density through effective pore filling and hydration product formation, while excessive amounts have detrimental effects due to weak phases and increased porosity.

## 4. Examination of the Microscopic Operation Mechanism

### 4.1. XRD Analysis

**Figure 9:**
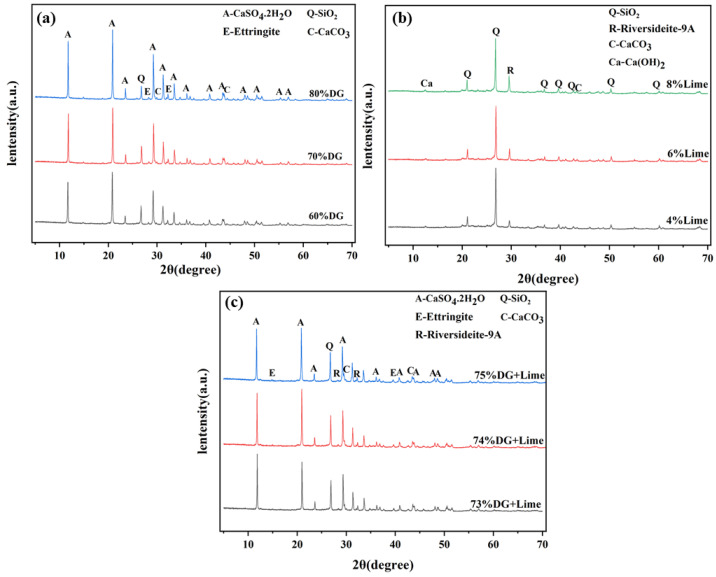
XRD spectra of the (**a**) CF system, (**b**) CL system, and (**c**) CFL system.

To investigate the effects of desulfurized gypsum and lime on the hydration products and microstructure of coal gangue-based systems, XRD technique (XRD) analysis was conducted on the CF, CL, and CFL systems. Figure 9 presents the XRD spectra, followed by a discussion of the observed mineral phases and their formation mechanisms, along with their contributions to the material properties.

In the CF system, the main mineral phases include gypsum (CaSO_4_·2H_2_O), quartz (SiO_2_), calcite (CaCO_3_), and ettringite (Ca_6_Al_2_(SO_4_)_3_(OH)_12_·26H_2_O). As shown in Figure 9a, at 70% DG content, ettringite, and calcite peaks are most prominent, indicating that sulfate ions (SO_4_^2−^) and alumina phases (Al_2_O_3_) from coal gangue react to form large amounts of ettringite. In an alkaline environment, calcium sulfate in DG releases SO_4_^2−^ ions that react with alumina from coal gangue, promoting ettringite formation. This reaction fills the micro-pores, improving early strength and enhancing the material’s volume stability. When the DG content is below or above 70%, insufficient or excessive unreacted gypsum leads to reduced material strength due to a lack of hydration products.

In the CL system, the primary mineral phases include quartz (SiO_2_), riversideite-9Å (Ca_5_Si_6_O_16_(OH)_2_·H_2_O), calcium hydroxide (Ca(OH)_2_), and calcite (CaCO_3_).As illustrated in Figure 9b, at 6% lime content, riversideite-9Å peaks are most pronounced, indicating an optimal reaction between Ca^2+^ ions from lime and the silicate phases (SiO_2_) in coal gangue, leading to the formation of calcium silicate hydrate (C-S-H), crucial for strength development. In this mechanism, Ca^2+^ ions from lime react with active silica and alumina in coal gangue, forming C-S-H and AFt, which densifies the microscopic structure and enhances mechanical properties.

In the CFL system, the synergistic effect of gypsum and lime promotes the formation of both ettringite and calcium silicate hydrates. As depicted in Figure 9c, at 4% lime content, riversideite-9Å and ettringite peaks are most distinct, indicating efficient reactions among Ca^2+^, SO_4_^2−^, and active alumina and silica phases in coal gangue. Ca^2+^ ions from lime facilitate the formation of C-S-H and also enhance the reaction between sulfate ions and alumina, promoting ettringite formation. These hydration products fill the micro-pores, significantly improving the material’s microstructural integrity. The synergistic effect of gypsum and lime also optimizes the pore structure and reduces carbonation, contributing to long-term stability [50].

Through XRD analysis and mechanism explanation, the three systems—CF, CL and CFL—exhibit distinct differences in mineral phase formation and reaction mechanisms. In the CF system, 70% DG is optimal for maximizing ettringite formation, which plays a critical role in strength development. In the CL system, 6% lime facilitates the most favorable formation of stable C-S-H. The CFL system, with 4% lime and 70% DG, shows the best performance, where the synergistic action between gypsum and lime enhances the generation of AFt and the formation of C-S-H, significantly improving physical-mechanical characteristics and microstructural stability. The combination of DG and lime accelerates hydration product formation, refines the microstructure, and reduces porosity, thus enhancing strength and durability [51].

### 4.2. SEM Observation

**Figure 10:**
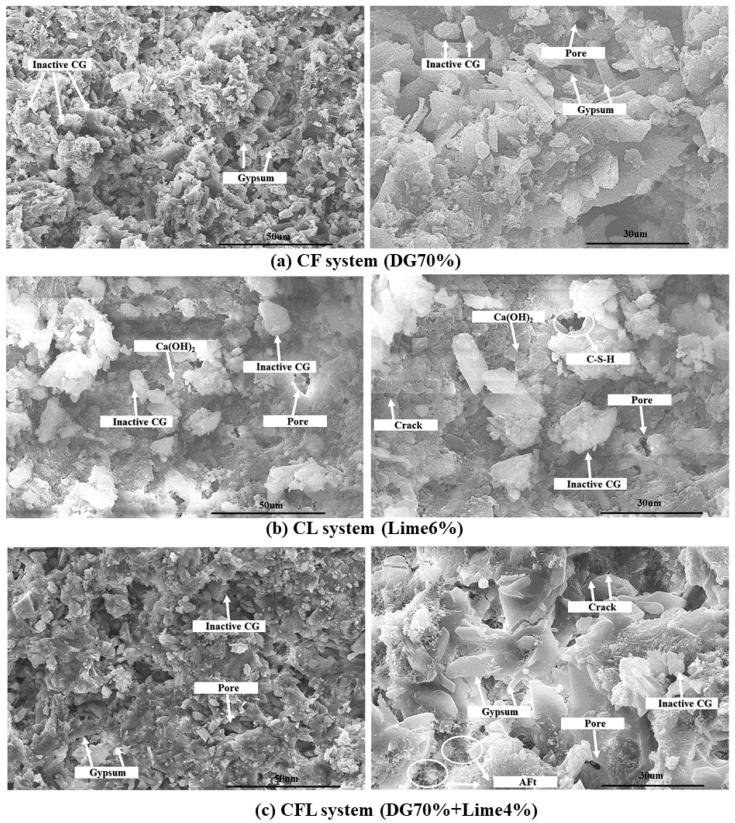
SEM images of the (**a**) CF system (DG 70%), (**b**) CL system (lime 6%), and (**c**) CFL system.

To investigate the microstructural characteristics of coal gangue-based composites with varying contents of desulfurized gypsum and lime, scanning electron SEM analysis was conducted on the CF (DG 70%), CL (lime 6%), and CFL (DG 70% + lime 4%) systems. The SEM images (Figure 10a–c) reveal significant differences in the morphology, porosity, and distribution of hydration products among the three systems, which directly influence their mechanical properties and overall performance.

In the CF system (shown in Figure 10a), SEM images show that the primary components are unreacted coal gangue particles and calcium sulfate dihydrate (CaSO_4_·2H_2_O). The coal gangue particles exhibit irregular shapes with smooth surfaces, indicating minimal dissolution of active components. This suggests that the coal gangue in this system remains mostly inert, contributing little to the hydration reactions. In contrast, calcium sulfate dihydrate forms characteristic lamellar structures that are uniformly distributed throughout the matrix. These lamellar structures provide structural support by filling pores and enhancing matrix density. Despite the significant presence of unreacted coal gangue particles, the high gypsum content effectively fills matrix pores, resulting in a dense microstructure that contributes to the system’s maximum early-stage strength. The interwoven lamellar crystals of gypsum improve adhesion and provide stability, compensating for the lack of coal gangue reactivity.

SEM images of the CL system (shown in Figure 10b) with 6% lime show a microstructure composed of unreacted coal gangue particles, hexagonal plate-like calcium hydroxide (Ca(OH)_2_) crystals, and sparsely distributed calcium silicate hydrate (C-S-H) phases. The hexagonal Ca(OH)_2_ crystals, typical hydration products, indicate that pozzolanic reactions are present but limited due to the relatively low lime content. The unreacted coal gangue particles have a dense, glassy morphology, suggesting low reactivity, similar to that seen in the CF system. Additionally, a significant number of pores and microcracks are observed throughout the matrix, likely caused by incomplete packing and weak adhesion between lime and coal gangue particles. Despite these structural imperfections, the formation of Ca(OH)_2_ crystals helps fill matrix pores, and the limited C-S-H phases provide some degree of cohesion, resulting in the system’s maximum strength at 6% lime content. However, the low reactivity of coal gangue and high porosity suggest that the material’s compactness is not optimal, and further activation may be required to fully utilize the pozzolanic potential of coal gangue.

The SEM analysis of the CFL system (shown in Figure 10c), which combines coal gangue, lime, and desulfurized gypsum, reveals a more complex microstructure compared to the CF and CL systems. The gypsum forms characteristic lamellar structures, similar to those in the CF system, but in this case, they are accompanied by needle-like ettringite (AFt) crystals, resulting from the reaction between calcium hydroxide, sulfate ions, and alumina from coal gangue. These ettringite crystals contribute to early-stage strength and volume stability. However, a large proportion of unreacted coal gangue particles is still present, indicating limited reactivity under the current conditions. Additionally, numerous pores and microcracks are visible throughout the matrix, which may be attributed to incomplete packing and shrinkage during hydration. These structural imperfections reduce the compactness of the system, limiting its mechanical properties. While the gypsum’s filler effect and the generation of ettringite helps to enhance early strength, the high porosity and unreacted coal gangue suggest that the CFL system has not fully utilized the reactivity of its components, and further modifications, such as increasing lime content, may be necessary to achieve a denser and more reactive microstructure.

The SEM analysis of the CF, CL and CFL systems highlights distinct differences in microstructural development and hydration product distribution, closely related to their compositions [52]. In the CF system, the high gypsum content provides effective pore filling and enhances matrix density through the formation of lamellar calcium sulfate dihydrate crystals. This compensates for the low reactivity of coal gangue, resulting in high early-stage strength. In contrast, the CL system with its low lime content shows limited formation of hydration products, leading to high porosity and microstructural defects, although the hexagonal Ca(OH)_2_ crystals help fill pores and contribute to strength. The CFL system benefits from the combined effects of gypsum and lime, initiating the concurrent genesis of gypsum lamellae and ettringite, which enhance the strength to some degree. However, the presence of unreacted CG and high porosity indicates that the system’s reactivity is low, and further activation is required to achieve a denser, more homogeneous microstructure [53].

### 4.3. TGA/DTG Analysis

**Figure 11:**
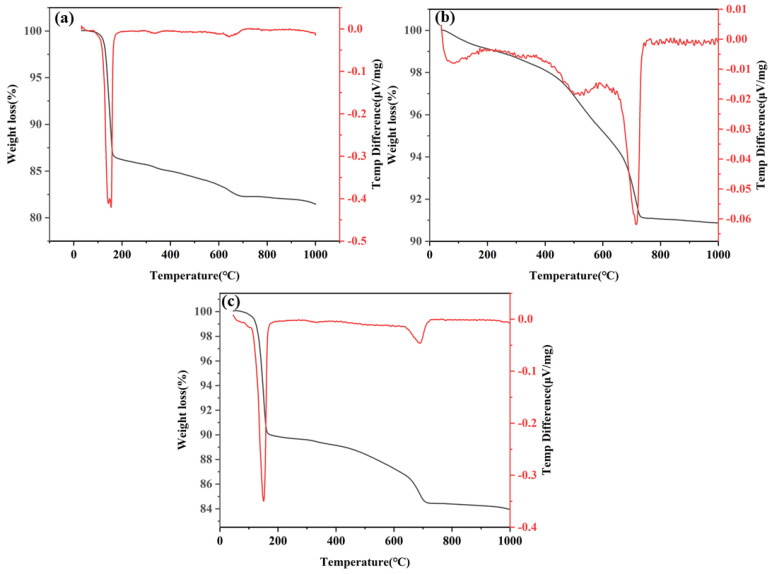
TGA and DTG curves of the (**a**) CFL system (DG 70%), (**b**) CFL system (lime 6%), and (**c**) CFL system (DG 70% + lime 4%).

Figure 10 shows the TGA and derivative results, with the corresponding curves presenting the differential mass of CF (DG 70%), CL (lime 6%) and CFL (DG 70% + lime 4%) system composite samples at 28 days of hydration. The curves are shown in Figure 11a, Figure 11b, and Figure 11c for the CF, CL, and CFL systems, respectively.

The TGA curve for the CF system, shown in Figure 11a, shows an initial weight loss of up to 150 °C, primarily due to the evaporation of water from gypsum in the DG. The dehydration of gypsum leads to the release of water, which is reflected in a notable weight loss in this low-temperature range [54]. From 150 °C to 250 °C, further weight loss happens, correlating with the dehydration of C-S-H gel’s interlayer water and the breakdown of minor hydration products [55]. A sharp mass loss is observed between 450 °C and 600 °C, which results from the decomposition of C-S-H gel, C_2_ASH_8_, and C_3_ASH_6_. Around 675 °C, another weight loss is seen, indicating the decarbonization of CaCO_3_ [56].

The TGA curves for both the CL and CFL systems, shown in Figure 11b,c, exhibit similar trends in thermal behavior. Initially, both systems show weight loss up to 150 °C due to water evaporation. In the range of 150 °C to 250 °C, gradual weight loss occurs, attributed to the dehydration of C-S-H gel and minor hydration phases. A significant mass loss is observed around 470 °C, corresponding to the decomposition of CH into CaO and water, indicating extensive hydration processes.

While both systems exhibit decarbonization of calcium carbonate around 675 °C, the CFL system, shown in Figure 11c, shows reduced overall weight loss compared to the CL system. This reduction suggests enhanced pozzolanic reactions, as the interaction between coal gangue, gypsum, and lime in the ternary system accelerates hydration and reduces residual CH content. The synergistic effects of gypsum and lime in the CFL system contribute to more efficient thermal behavior and hydration compared to the CL system.

These TGA/DTG curves collectively demonstrate the thermal stability and decomposition characteristics of the CF, CL and CFL systems, with notable phase changes occurring at distinct temperature ranges related to hydration and pozzolanic activity.

### 4.4. NMR Analysis

In silicate mineral structures, each Si atom is usually surrounded by four O atoms, generating a (SiO_4_)^4−^ tetrahedron. This tetrahedron serves as the basic structural building block of silicates. In ^29^Si NMR [57] testing and analysis, different ^29^Si NMR signals represent the degree of aggregation of the silicon-oxygen tetrahedra.

**Figure 12:**
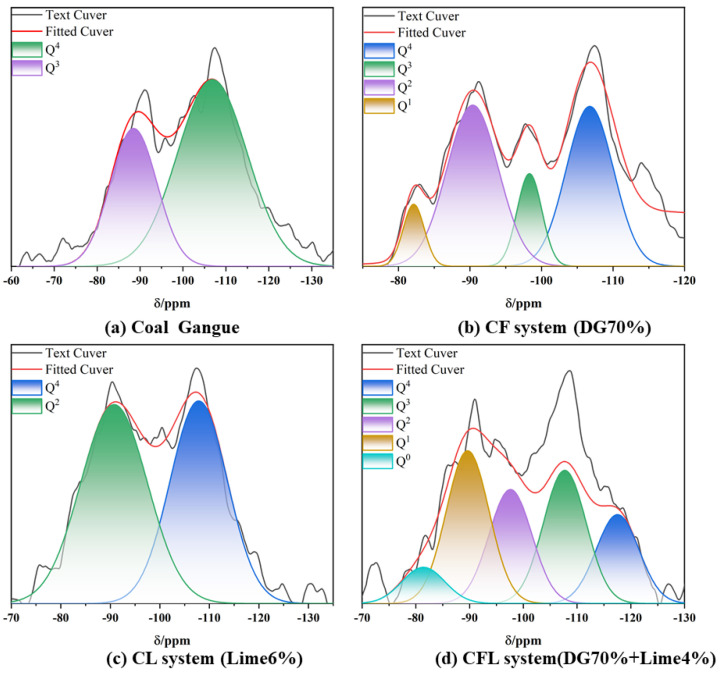
^29^Si NMR spectra of (**a**) coal gangue, (**b**) the CF system, (**c**) the CL system, and (**d**) the CFL system.

As shown in Figure 12a, the peaks of absorption in the CG raw material primarily originate from the Q^3^ structural units of kaolinite (−93.59 ppm) and the Q⁴ structural units of quartz (−110.14 ppm) [57].

As shown in Figure 11b and Table 4, Compared to coal gangue, the introduction of flue gas desulfurization (DG) gypsum in the CF system significantly alters the silicon aggregation structure within the coal gangue. Specifically, the proportion of Q^4^ units decrease, and new Q^1^ and Q^2^ units can be found in Figure 11. This shows in the presence of Ca^2+^ [58], the silicon-oxygen structure of the coal gangue undergoes depolymerization and reorganization, forming more amorphous structures and C-S-H gel. Flue gas desulfurization gypsum (primarily composed of CaSO_4_·2H_2_O) releases Ca^2+^ ions under alkaline conditions, which interact with Si-O bonds, breaking the original silicon-oxygen network. This process leads to the transition of silicon coordination from Q^4^ to Q^2^ and Q^1^ states, as demonstrated in Figure 12b [59].

Figure 12c presents the ^29^Si NMR spectrum of the CL system, showing two main silicon-oxygen structures, Q^4^ and Q^2^. The Q^4^ peak, around −110 ppm, represents the presence of silicon atoms bonded to four oxygen atoms, indicating a relatively ordered silicate network, such as quartz. Despite the addition of lime, this structural unit remains prominent, suggesting that a significant portion of the silicate framework remains intact. On the other hand, the Q^2^ peak, around −90 ppm, corresponds to silicon atoms bonded to two oxygen atoms, reflecting the partial depolymerization of the silicate network. This depolymerization is driven by the interaction of Ca^2+^ ions from the lime with Si-O bonds [60,61], resulting in the generation of C-S-H gel. The presence of both Q^4^ and Q^2^ units highlights the role of Ca^2+^ in breaking down and reorganizing the silicon-oxygen network while retaining a portion of the original silicate structure, as observed in Figure 12c.

Figure 12d is the ^29^Si NMR spectrum of the CFL system, demonstrating multiple silicon-oxygen coordination structures, including Q^4^, Q^3^, Q^2^, Q^1^, and Q^0^. These represent the significant changes in the silicon-oxygen tetrahedra as a result of the interactions between coal gangue, gypsum, and lime. The Q^4^ peak around −110 ppm indicates the presence of intact silicon-oxygen tetrahedra, likely from unreacted coal gangue or quartz. The Q^3^ and Q^2^ peaks, near −100 ppm and −90 ppm respectively, suggest partial depolymerization, forming amorphous C-S-H gel. The Q^1^ and Q^0^ peaks, corresponding to further depolymerization, indicate the breakdown of the silicon-oxygen network under the influence of Ca^2+^ ions from the gypsum and lime. This spectrum, shown in Figure 12d, reflects the extensive structural reorganization within the system, driven by the synergistic effects of gypsum and lime, leading to increased reactivity and the formation of various silicate phases.

## 5. Discussion

In this study, we explored the synergistic effect of DG and lime in the CFL system and their impact on the hydration characteristics and mechanical properties of coal gangue-based materials. The results indicated that the combination of DG and lime significantly enhanced the hydration reaction in the CFL system, promoting the formation of key hydration products, which improved the material’s early strength and long-term durability.

1. Synergistic Effect of DG and Lime

In the CFL system, lime serves as a rich source of Ca^2+^ ions, while SO_4_^2−^ ions from DG react with Ca^2+^ ions from lime to facilitate the formation of hydration products—needle-like ettringite (AFt). The formation of AFt not only significantly enhances the early strength of the material but also, due to its expansive nature, aids in filling microcracks and increasing the material’s compactness. Compared to systems utilizing only lime or DG, the formation of AFt in the CFL system was more pronounced, as evidenced by scanning electron microscopy (SEM) images that displayed well-developed needle-like AFt crystals embedded in the matrix. Furthermore, the SO_4_^2−^ ions from DG also promote the transformation of aluminosilicate phases in coal gangue, generating stable C-S-H (calcium silicate hydrate) and AFt phases. This process contributes to enhancing the overall mechanical performance and durability of the material, particularly during long-term hydration. Our experimental results align with similar studies in the literature, which have demonstrated that an adequate supply of Ca^2+^ and SO_4_^2−^ promotes AFt crystallization, thereby improving the hydration reaction and increasing material density [40,41,42,43].

2. Comparison with Existing Research

Our results indicate that under the activation of diatomaceous earth (DG) and lime, the primary hydration products in the coal fly ash (CFL) system are calcium silicate hydrate (C-S-H) and ettringite (AFt). This finding aligns with previous research on DG-activated coal gangue systems, which also observed that sulfate ions (SO_4_^2−^) promote AFt formation, thereby enhancing early strength (Relevant Study [3]). Compared to other coal gangue-based cementitious materials, the microstructure of the CFL system is denser and exhibits superior mechanical performance, which may be attributed to the synergistic effects of DG and lime. Additionally, our study found that the ratio of DG to lime plays a critical role in the formation of hydration products. In this study, the optimal ratio was determined to be 70% DG and 4% lime, which maximized the formation of AFt and C-S-H, significantly improving the material’s strength. This finding is consistent with other literature regarding the optimization of activator ratios (Relevant Study [4]).

3. Limitations and Future Directions

Although this study has made significant progress in understanding the hydration characteristics and mechanical properties of coal gangue activated by DG and lime, several limitations remain. First, despite the effectiveness of lime and DG in promoting coal gangue hydration, some coal gangue particles do not fully react, resulting in lower reactivity. This may adversely affect the material’s final performance, particularly during the early hydration stage. Scanning electron microscopy (SEM) analysis revealed that some coal gangue particles are encapsulated within the hydration products, thereby reducing their effective contact with calcium ions (Ca^2+^) and sulfate ions (SO_4_^2−^), which may be a contributing factor to their low reactivity.

Future research could focus on mechanical activation (e.g., finer grinding of coal gangue) to increase its specific surface area and enhance reactivity. Additionally, methods such as high-temperature pre-treatment or alkali activation may further promote the dissolution of aluminosilicate phases in coal gangue, improving its hydration reactivity.

4. Improvements and Optimization Suggestions

Based on the findings of this study, several potential improvements could be explored in future work:

Exploring Other Activators: in addition to DG and lime, the use of alkali activators (e.g., NaOH, KOH, or sodium silicate) could further promote the hydration reaction of coal gangue, increasing the formation of C-S-H and other hydration products.

Optimizing Curing Conditions: increasing the curing temperature (e.g., 40–60 °C) could accelerate the hydration reaction, improving coal gangue reactivity and mechanical performance.

Adjusting Particle Size and Optimizing Ratios: reducing the particle size of coal gangue and optimizing the DG to lime ratio could further enhance the hydration efficiency and mechanical properties of the material.

## 6. Conclusions

This study investigates the synergistic effects of flue gas desulfurization (DG) gypsum and lime in activating the reactivity of coal gangue and their application in environmentally friendly cementitious materials. By designing three different systems: the CF system, the GL system, and the CFL system, the impact of varying DG gypsum and lime content on the reactivity of coal gangue was systematically examined. Based on the experimental results, the following conclusions can be drawn:

(1) The introduction of DG gypsum significantly increases the early strength of the CF system. Its high reactivity promotes the formation of hydration products within the cementitious materials, enhancing the mechanical properties. An optimal amount of DG gypsum can improve the early strength, which is crucial for the initial phase of cementitious material performance.
(2) The addition of lime effectively improves the long-term strength of the CF system. The calcium ions provided by lime react with other hydration products to form more stable hydrates, contributing to long-term durability.
(3) In the CFL system, the combined effect of DG gypsum and lime further optimizes the cementitious properties of coal gangue. This system not only achieves superior mechanical performance but also shortens the setting time, demonstrating the synergistic effects of DG gypsum and lime on coal gangue reactivity.
(4) Microstructural analysis revealed the mechanism of coal gangue activation by DG gypsum and lime. The analyses show that the optimized microstructure enhances the material’s density and crack resistance, leading to improved overall performance.

In summary, by rationally adjusting the substitution ratios of DG gypsum and lime, this study provides valuable insights into the high-value utilization of coal gangue. It lays the experimental foundation for developing environmentally friendly cementitious materials, showcasing the sustainable application potential of these materials in the construction industry.

## Figures and Tables

**Table 1:** The chemical ingredient profiles of raw materials.

Raw Materials	SiO_2_	Al_2_O_3_	Fe_2_O_3_	MgO	K_2_O	CaO	MoO_3_	SO_3_
CG	39.876	34.921	10.414	1.311	3.841	4.793	1.183	0.088
DG	0.806	1.275	-	1.166	0.211	57.521	-	37.272
Lime	-	0.287	-	0.575	0.697	97.076	-	0.216

**Table 2:** Characteristics of the physical nature of raw materials.

Sample	Specific Gravity	Particle Size Parameters			
		D10/μm	D50/μm	D90/μm	STD/μm
CG	2.3	1.716	9.674	33.573	5.309
DG	2.4	2.170	25.605	61.150	9.830
Lime	2.6	1.542	8.022	53.713	8.695

**Table 3:** Experimental mix ratio.

	Sample	CG	DG	Lime	Water/Cement Ratio
CF system	1	90	10	0	0.35
	2	80	20	0	0.35
	3	70	30	0	0.35
	4	60	40	0	0.35
	5	50	50	0	0.35
	6	40	60	0	0.35
	7	30	70	0	0.35
	8	20	80	0	0.35
	9	10	90	0	0.35
CL system	10	98	0	2	0.35
	11	96	0	4	0.35
	12	94	0	6	0.35
	13	92	0	8	0.35
	14	90	0	10	0.35
CFL system	15	29	70	1	0.35
	16	28	70	2	0.35
	17	27	70	3	0.35
	18	26	70	4	0.35
	19	25	70	5	0.35
	20	24	70	6	0.35

**Table 4:** Relative peak area of the fitted curve of ^29^Si NMR spectra (%).

Sample	Q^0^	Q^1^	Q^2^	Q^3^	Q^4^
Coal gangue	0	0	0	28	72
CF system	0	10	30	25	35
CL system	0	0	40	0	60
CFL system	5	10	20	25	40

## Data Availability

The data are contained within the article.
